# UV-Protected Polyurethane/*f*-Oil Fly Ash-CeO_2_ Coating: Effect of Pre-Mixing *f*-Oil Fly Ash-CeO_2_ with Monomers

**DOI:** 10.3390/polym13193232

**Published:** 2021-09-23

**Authors:** Mohammad Mizanur Rahman, Md. Hasan Zahir, Aasif Helal, Rami K. Suleiman, Bashirul Haq, A. Madhan Kumar

**Affiliations:** 1 Interdisciplinary Research Center for Advanced Materials, King Fahd University of Petroleum & Minerals, Dhahran 31261, Saudi Arabia; ramismob@kfupm.edu.sa (R.K.S.); madhankumar@kfupm.edu.sa (A.M.K.); 2 Interdisciplinary Research Center for Renewable Energy and Power Systems (IRC-REPS), King Fahd University of Petroleum & Minerals, Dhahran 31261, Saudi Arabia; hzahir@kfupm.edu.sa; 3 Interdisciplinary Research Center for Hydrogen and Energy Storage (IRC-HES), King Fahd University of Petroleum & Minerals, Dhahran 31261, Saudi Arabia; aasifh@kfupm.edu.sa; 4 College of Petroleum Engineering and Geosciences, King Fahd University of Petroleum & Minerals, Dhahran 31261, Saudi Arabia; bhaq@kfupm.edu.sa

**Keywords:** waterborne polyurethane, degradation, coating

## Abstract

A series of UV-protected coatings were prepared using cerium-oxide-functionalized oil fly ash (*f*-OFA-CeO_2_) in waterborne polyurethane (WBPU) dispersions. Three monomers, namely, poly(tetramethyleneoxide glycol) (PTMG), polydimethylsiloxane-hydroxy terminated (PDMS) and 4,4-dicyclohexylmethane diisocyanate (H_12_MDI), were used to pre-mix with *f*-OFA-CeO_2_ separately, followed by the synthesis of WBPU/*f*-OFA-CeO_2_ dispersions. The *f*-OFA-CeO_2_ distribution and enrichment into any part (top/bottom/bulk) of the coating was strongly affected by the pre-mixing of *f*-OFA-CeO_2_. The *f*-OFA-CeO_2_ was densely distributed in the top, bottom and bulk when the *f*-OFA-CeO_2_ was pre-mixed with PDMS, H_12_MDI and PTMG, respectively. Only an *f*-OFA-CeO_2_-enriched top surface showed excellent UV protection. The lowest UV-degraded exposed coating was found when the top surface of the coating was *f*-OFA-CeO_2_-enriched.

## 1. Introduction

There is a wide range of organic coatings available commercially. Each coating has both advantages and disadvantages. Moreover, each coating has been commercialized for certain application purposes. It is important to choose the proper coating before applying it to a surface, otherwise the coating may fail to fulfill the desired purpose. The coating itself can be divided into three parts, namely, bottom, bulk and top layers. The top part is very important for outdoor applications, as this part is mainly associated with UV degradation and discoloration, as well as functioning as a barrier for electrolyte passage [1,2,3,4,5].

Polyurethane (PU) coatings are well known for their excellent protective properties. PU coatings are widely used in both indoor and outdoor applications. In particular, their self-healing nature, sufficient mechanical strength and excellent barrier resistance make PU coatings a highly valuable option in many outdoor applications [2,3,6].

However, PU coatings considered for outdoor applications still encounter challenges regarding UV rays [2,7,8]. Coatings often degrade under UV rays. Recent research proved that UV degradation can be delayed when proper nanoparticles and organic compounds are used [8,9,10]. However, the use of nanoparticles is challenging, as PU coatings have the tendency to sacrifice their mechanical strength and adhesive strength in the presence of improper or excessive amounts of nanoparticles [7,8]. Moreover, long-term stable PU/nanocomposite dispersion is not easily achievable [11,12]. Many researchers have sought to overcome these challenges by developing new techniques. Different carbon-based nanoparticles have shown very promising results in this regard; however, critically, they are associated with many issues [2]. It is not straightforward to address these challenges in traditional PU coatings.

OFA is a byproduct generated by power and desalination plants in Saudi Arabia [8]. OFA is mainly a carbon (90%)-based spherical particle [8,13]. The local OFA has very limited uses; it is mainly used in the construction sector in very limited quantities. OFA is mainly used for damping in open land. OFA thus presents a considerable burden not only for the government but also for the environment, representing a major threat to ecosystems. Recently, a few researchers [8,13,14] in Saudi Arabia have demonstrated some other promising applications of OFA in organic coatings. OFA and functionalized OFA (*f*-OFA) can improve the thermal, mechanical and protective properties of polymers [8,13]. Our latest research study [8] also showed that cerium-based functionalized OFA (*f*-OFA-CeO_2_) can be used for UV protection, as well as corrosion protection. The attached functionalized metal oxide can absorb the UV rays and it opposes the polymer chain scission. Ultimately, the UV degradation of the coating slowed down significantly [8]. We also showed that in situ polymerization [15] is better than blending processes in order to achieve better protective properties using zinc-based functionalized OFA (*f*-OFA-ZnO). We believe that the *f*-OFA distribution in the coating can also play a vital role, leading to longer-term protection. In particular, the *f*-OFA distribution on the top surface can act as a prime defense for protection. UV rays can be absorbed in *f*-OFA; thus, the degradation of the coating can be delayed for a longer period of time.

Recently, waterborne polyurethane (WBPU) coating materials have attracted interest for a variety of outdoor coating applications. The main advantage of WBPU coatings is their environmental friendliness, as this coating material is dispersed mainly in water. To fulfill a range of needs, different monomers are also used in WBPU coating materials. The use of different nanoparticles is also common in WBPU coatings [7,8]. Functionalized nanoparticles are also used to further enhance the protective properties of coatings [7,8]. However, to enhance such protective properties, the distribution of functionalized nanoparticles in the top/bottom/bulk layer of the coating has not been considered yet. More specifically, there is no report on the effect of functionalized nanoparticle distribution on the protective properties based on their distribution on the coating layer. In this study, we used three different PU monomers, poly(tetramethyleneoxide glycol) (PTMG), Polydimethylsiloxane-hydroxy terminated (PDMS) and 4,4-dicyclohexylmethane diisocyanate (H_12_MDI), to pre-mix *f*-OFA-CeO_2_ to facilitate the nanoparticle distribution on the top surface in order to enhance the resistance of the material to UV degradation. To facilitate the distribution of *f*-OFA on the top surface, the use of PDMS in the coating might have been a good choice, because PDMS has low surface energy and it can move to the top surface easily. Unfortunately, PDMS is brittle and, thus, it is usually used along with other polyols. In our previous study [16], we showed the excellent protective properties achieved with 10.75 mol% PDMS along with 7.50 mol% PTMG; thus, we followed the same formulation to distribute *f*-OFA-CeO_2_ in WBPU dispersions. We pre-mixed *f*-OFA-CeO_2_ separately with PTMG, H_12_MDI, or PDMS. The mixed solution was used to prepare WBPU/*f*-OFA-CeO_2_ dispersions. The *f*-OFA-CeO_2_ distribution in the three parts of the coating (top, bottom and bulk) was assessed by XPS analysis. The dispersions were coated onto mild steel panels. The effect of *f*-OFA-CeO_2_ enrichment against UV degradation was considered for 8 months under real atmospheric conditions in Saudi Arabia.

## 2. Materials and Methods

The base monomers were collected from Sigma-Aldrich, St. Louis, MO, USA. The OFA was collected from the local Shuaibah power plant, Saudi Electricity Company, Jeddah, Saudi Arabia. The monomers, such as 4,4-dicyclohexylmethane diisocyanate (H_12_MDI), triethylamine (TEA) and ethylene diamine (EDA), were used after dehydration with 4 Å molecular sieves for seven days. Dimethylolpropionic acid (DMPA), cerium oxide and dibutyltindilaurate were used as received. Poly(tetramethyleneoxide glycol) (PTMG Mn = 2000) and Polydimethylsiloxane-hydroxy terminated (PDMS Mn = 550) were vacuum dried at 90 °C for three hours prior to use. The OFA functionalization was performed according to our previous report [8]. The pristine WBPU dispersion was also carried out according to our previous report [7,8].

### 2.1. Preparation of WBPU/f-OFA-CeO_2_ Dispersions (WBPU-PTMG-Ce, WBPU-PTMG-NCO-Ce, WBPU-PDMS-Ce and WBPU-PDMS-NCO-Ce)

An amount of 2.0 wt% *f*-OFA-CeO_2_ was pre-mixed with either PTMG, PDMS, or H_12_MDI (10.00 g). The mixture was mechanically stirred for 30 min at 60 °C. Following this, the mixture was ultrasonicated for 20 min. This mixture was then added to the prepolymer in another vessel. The prepolymer was prepared by charging the calculated amount of PTMG, PDMS, DMPA and H_12_MDI (see Table 1). The neutralization, dispersion and chain extension were performed according to our previous report [7]. The solid content of dispersion was around 30.0 wt%.

### 2.2. Substrate Coating

First, wet coatings, with the three defined thicknesses of 100, 200 and 300 μm, were prepared using the dispersions. Mild steel was used as a substrate. The wet coatings were dried at room temperature for 24 h. These dried coatings were further dried at 70 °C in an oven. Finally, completely dried coatings with thicknesses of approximately 30, 60 and 90 μm, from original 100, 200 and 300 μm wet coatings, respectively, were obtained. The coatings with 30 and 60 μm were free from delamination, coagulation and cracking. However, the coating with 90 μm had slightly different appearance. A few scattered small cracks had appeared on the coating. However, this cracking was not noticed after room-temperature drying for 24 h; the cracks appeared after oven-heating of the sample. The cracking appeared due to the evaporation of the remaining solvent trapped in the coating. Thus, the coating with 90 μm was not considered for this study. At the same time, we faced difficulties in analyzing the bulk of the 30 μm coating for XPS analysis. Therefore, only the wet coating with a 200 μm thickness was considered for this study. Currently, the challenges of drying coatings with different thicknesses is under investigation. Those results will be published in near future.

### 2.3. Exposure Test

All coatings were exposed to open atmosphere near the seaside in Jubail, Saudi Arabia. The coatings were exposed from 1 March to 30 October 2019. The coated specimens were kept in a rack and monitored by naked eye. The average high temperature and low temperature were 42 °C and 23.5 °C, respectively.

### 2.4. Characterization

All coatings were analyzed by FT-IR (Impact 400D, Nicolet, Madison, WI, USA) spectroscopy to confirm the completion of the reaction, as well as the synthesis of the expected WBPU coatings. The film hardness was measured by the Shore A (Shanghai Liuling Instrument Company, Shanghai, China) hardness test according to the ASTM D2240-75 specification. The results provided are the average of five tests. A Theta Optical tensiometer instrument (Attension, Helsinki, Finland) was used to measure the water contact angle. The respective film was fixed properly on a workbench; then, a drop of water (5 μL) was deposited by a micro syringe on the film and, immediately, the contact angle was measured. A total of five measurements was performed for each sample and the mean values were calculated. A UV–Vis spectrophotometer (UV-3600 plus, Shimadzu, Tokyo, Japan) was used to measure the UV absorption properties of the coatings. XPS (ESCA 250 XPS, Thermo Scientific, East Grinstead, UK) was performed for the three parts of all films, i.e., the top of the surface, bottom and mid-section. The film was prepared on a Teflon disc, in accordance with our previous report [8].

## 3. Results and Discussion

**Figure 1:**
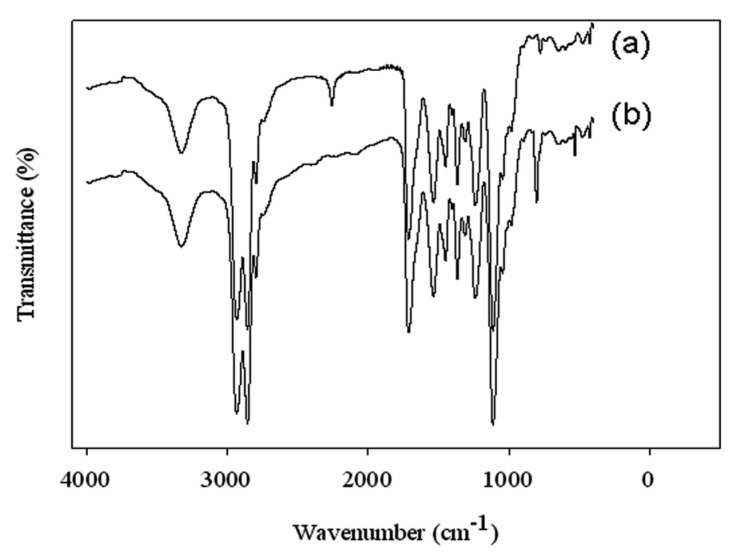
FT-IR spectra of reaction stages during preparation of WBPU-PDMS-Ce dispersion, (**a**) before adding *f*-OFA-CeO_2_ into prepolymer and (**b**) after completion of reaction between *f*-OFA-CeO_2_ and prepolymer followed by dispersion and chain extension.

To maintain the same stoichiometric ratio of monomers in the coatings, the monomer contents were fixed as follows: H_12_MDI 50.00, DMPA 23.00, polyol 18.25 and EDA 8.75 mol% (see Table 1). The *f*-OFA-CeO_2_ content was also fixed at 2.00 wt% for all *f*-OFA-CeO_2_-based coatings. The *f*-OFA-CeO_2_ content was taken as 2.00 wt% due to the excellent protective properties; this was achieved in our previous reports [8]. All the dispersions were prepared by following the prepolymer process. Although we applied pre-mixing of *f*-OFA-CeO_2_ with three different monomers, all the respective dispersions were stable, without any precipitation. The FT-IR spectra (Figure 1) of the dispersions also showed similar identical bands, confirming that all the dispersions had been properly prepared. It was also confirmed that the pre-mixing of *f*-OFA-CeO_2_ did not hinder the preparation of the dispersions. A band at 560 cm^−1^ in the *f*-OFA-CeO_2_-based films also confirmed the presence of *f*-OFA-CeO_2_ in the dispersions. Moreover, an identical peak at 806 for Si-CH_3_ also appeared for all PDMS-based dispersions, confirming that the PDMS-*f*-OFA-CeO_2_ had properly reacted with the NCO group. The attached OH group in PDMS-*f*-OFA-CeO_2_ reacted with the NCO group of prepolymer. Eventually, the band at 2170 cm^−1^ disappeared and the peak for CeO_2_ and siloxane appeared (see Figure 1). Thus, the PDMS-*f*-OFA-CeO_2_ mixture had no detrimental effect on the synthesis of the polymer.

**Figure 2:**
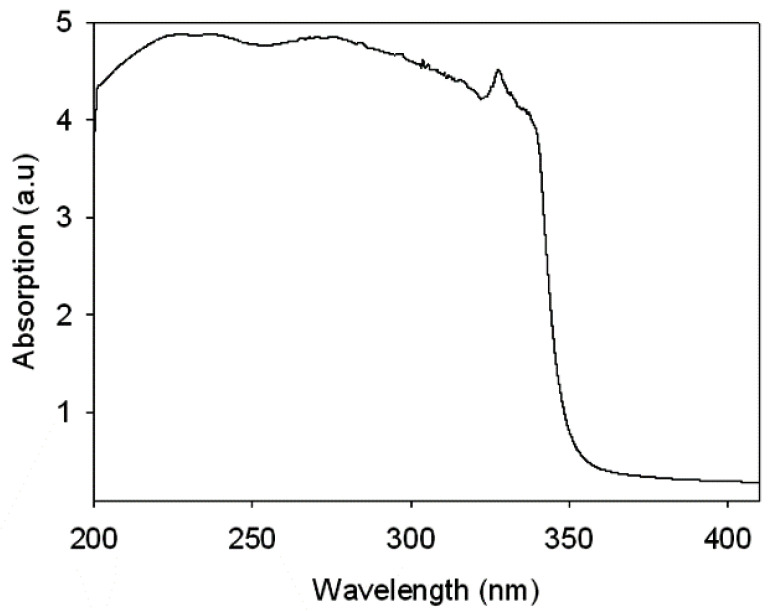
UV spectrum of WBPU-PDMS-Ce film.

UV–Vis spectroscopy is a good technique to assess the initial suitability of a coating material under outdoor UV exposure conditions. A typical UV–Vis spectrum is shown in Figure 2. As expected, all coatings except pristine dispersions showed a broad peak at around 280–400 nm. The peak appeared due to the presence of *f*-OFA-CeO_2_. The peak appeared almost at the same position and with the same intensity. This confirmed that the polyol altering, or mixing process did not interfere with the UV absorption by *f*-OFA-CeO_2_. All coatings except the pristine WBPU coatings (WBPU-PTMG and WBPU-PDMS) can be used in outdoor applications, including under UV-exposure conditions.

Polymer stiffness was measured with the hardness test. The test values are summarized in Table 1. It is well known that the stiffness depends on the polymer structure, such as linear, crosslink or hyperbranched, as well as on its plasticity [17]. The hardness was affected by the addition of *f*-OFA-CeO_2_. The hardness increased greatly with 2.0 wt% *f*-OFA-CeO_2_. The increased value is an indication of a compact structure by addition of *f*-OFA-CeO_2_. Therefore, the coating changed to a strong compact structure by the 2.0 wt% addition of *f*-OFA-CeO_2_. The *f*-OFA-CeO_2_ worked as a reinforcement filler and anchored strongly with a polymer chain; ultimately, the hardness increased.

**Figure 3:**
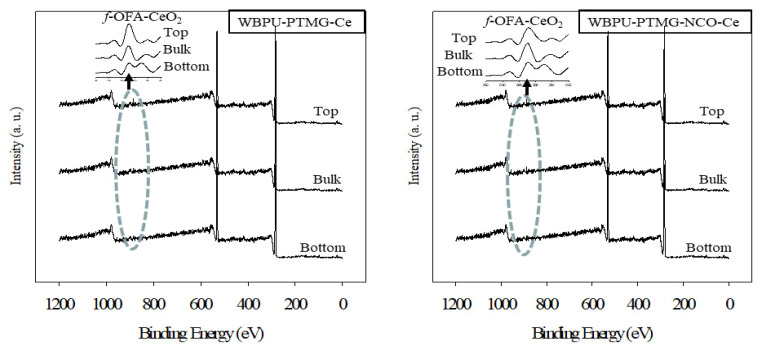
XPS scan of WBPU-PTMG-Ce and WBPU-PTMG-NCO-Ce films.

**Figure 4:**
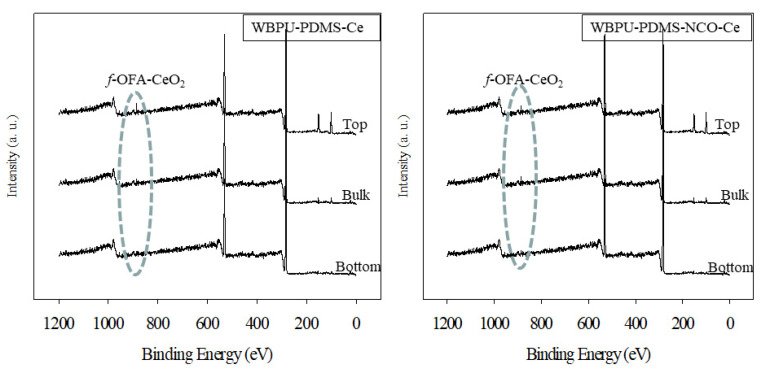
XPS scan of WBPU-PDMS-Ce and WBPU-PDMD-NCO-Ce films.

**Figure 5:**
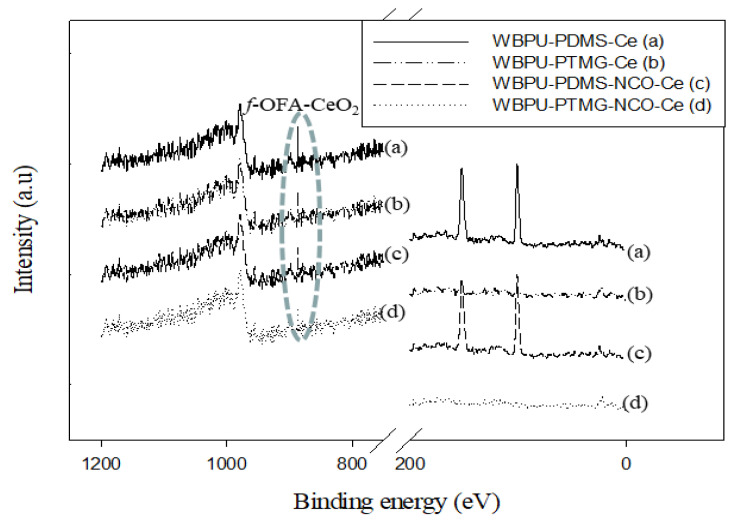
XPS scan of top surface of typical coatings.

The XPS results are shown in Figure 3, Figure 4 and Figure 5. All coatings showed typical peaks at 531 (oxygen (1s)), 402 (nitrogen (1s)) and 285 eV (carbon (1s)). Two new peaks at 101 (silicon (1s)) and 150 eV (silicon (2p)) for the siloxane group appeared for all PDMS-based coatings. There were no significant changes in the peaks of oxygen, nitrogen and carbon. The peaks were almost in the same position and had almost the same intensity.

XPS is a very useful technique to evaluate the *f*-OFA-CeO_2_ enrichment in any part of the coating (either top surface, bottom, or bulk). As our investigation was focused on the enrichment of *f*-OFA-CeO_2_, we mainly focused on a particular peak at 887 eV for CeO_2_ [8]. Any peak at 887 eV confirmed the presence of *f*-OFA-CeO_2_. A higher intensity of this peak also confirmed a higher amount of *f*-OFA-CeO_2_ distribution. Though the peak at 887 eV appeared in all three parts (top, bottom and bulk) for all *f*-OFA-CeO_2_-based coatings, the intensity was clearly different in the three different sections of each coating (see Figure 3 and Figure 4). For the polyol (PTMG or PDMS) and *f*-OFA-CeO_2_ mixed coatings, a high and intense peak was recorded on top of the surface (see Figure 5). At the same time, a weak peak appeared in the bulk and a very light peak was found in the bottom section (see Figure 3 and Figure 4). This implies that the *f*-OFA-CeO_2_ mostly moved onto the top surface due to the mixing process. The most interesting result was found when we compared the peaks for PTMG and PDMS polyol-based coatings (see Figure 5). The peak at the top surface was clearly much more intense for PDMS than that for PTMG. The lower surface energy of PDMS might have contributed to the movement of *f*-OFA-CeO_2_ mostly to the top of the surface along with PDMS. For the H_12_MDI and *f*-OFA-CeO_2_ pre-mixed coatings (WBPU-PDMS-NCO-Ce and WBPU-PTMG-NCO-Ce), the peaks were almost the same at the top, bottom and bulk; this implies that the *f*-OFA-CeO_2_ was almost equally distributed at the top, bottom and bulk. The *f*-OFA-CeO_2_ distribution was not affected by the addition of PDMS polyol. When all the best-performing coatings were compared, it was clear that the WBPU-PDMS-Ce coating was the most enriched with *f*-OFA-CeO_2_.

Many outdoor coatings suffer due to their lower hydrophobicity and, thus, water enters inside the coating, causing delamination. Thus, a hydrophobic coating is always preferable in outdoor applications. A water contact angle test was completed to verify the hydrophobicity of the coatings. The results are summarized in Table 1. The hydrophobicity changed with the change in polyols, CeO_2_ presence and *f*-OFA-CeO_2_ mixing methods. The water contact angle values followed the order WBPU-PDMS-Ce < WBPU-PDMS-NCO-Ce < WBPU-PTMG-Ce < WBPU-PTMG-NCO-Ce < WBPU-PDMS < WBPU-PTMG. As expected, higher values were recorded for PDMS-based coatings than for PTMG-based coatings. The Si-O-Si group led to higher values for the PDMS-based coatings. The water contact angle was always higher for the *f*-OFA-CeO_2_-based coatings than for the pristine WBPU coating. The *f*-OFA-CeO_2_ also increased the hydrophobicity, although the improvement was not the same for all the coatings. The highest level of improvement was found using PDMS, as well as when the *f*-OFA-CeO_2_ was pre-mixed with PDMS prior to polymerization. The initially improved hydrophobicity can be ascribed to the barrier effect of the *f*-OFA-CeO_2_. By the addition of *f*-OFA-CeO_2_ into the coating, the resin of the coating was stiffer; this stiff structure resisted water penetration. This was reflected by higher contact angle values. The pre-mixed *f*-OFA-CeO_2_ and PDMS also contributed to the improved hydrophobicity by distributing the particles mostly on top of the surface. As the *f*-OFA-CeO_2_ particles were distributed at the maximum using this method (confirmed by XPS), the top surface was highly enriched with *f*-OFA-CeO_2_. This made water penetration difficult; thus, the WBPU-PDMS-Ce coating further shifted to a higher contact angle value. Among all the coatings, the WBPU-PDMS-Ce showed the highest value due to the PDMS group, when the *f*-OFA-CeO_2_ was pre-mixed with PDMS.

**Figure 6:**
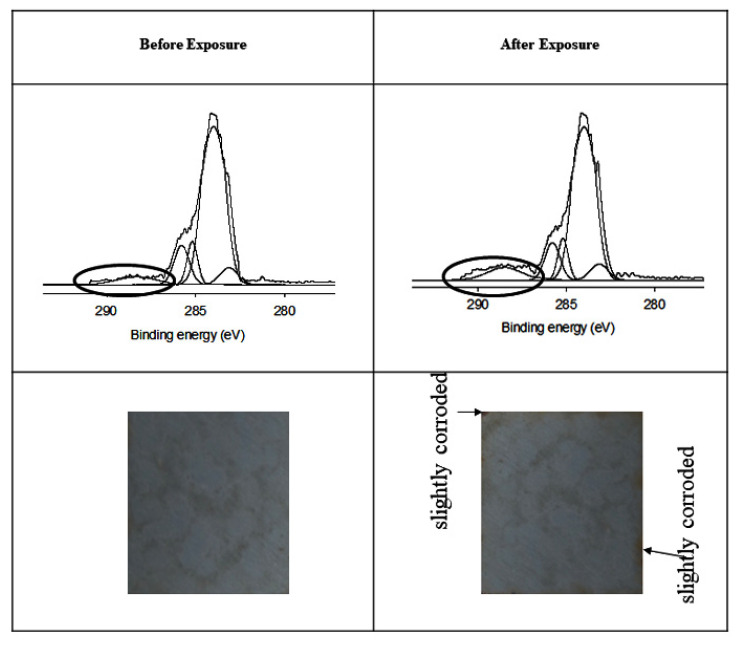
Photograph and XPS scan of exposed WBPU-PDMS-Ce coating.

All coatings were assessed for their feasibility as outdoor UV-resistant coatings. The coatings were exposed near the seaside for 8 months. The WBPU-PTMG and WBPU-PDMS coatings were completely corroded (not shown). This occurred because the coatings were degraded and they had no resistance to electrolyte passage. The coatings of WBPU-PTMG-Ce, WBPU-PTMG-NCO-Ce and WBPU-PDMS-NCO-Ce were also moderately corroded. This implies that the addition of *f*-OFA-CeO_2_ can only slightly improve the resistance to UV degradation for the mentioned coatings. As the top surface was not enriched with *f*-OFA-CeO_2_ for these coatings, the coatings degraded and were ultimately corroded. Only one exposed coating, WBPU-PDMS-Ce, appeared unaffected visually, without any cracking, delamination, or corrosion (see Figure 6). The *f*-OFA-CeO_2_-enriched surface resisted degradation; thus, an almost uncorroded specimen was found. To elucidate this finding, the exposed WBPU-PDMS-Ce coating was further analyzed via XPS (see Figure 6). It was shown that the peak at 289 eV appeared from the chain secession. Due to the carboxylate group in the WBPU coating, a peak in a similar area also appeared [8]. Thus, a high and intense peak at this position indicates the degradation of the coating [8]. In the deconvoluted curves, a very narrow and intense peak was seen. This also confirmed that very minor coating degradation occurred during this time.

## 4. Conclusions

CeO_2_-functionalized waste material OFA (*f*-OFA-CeO_2_) was used to enhance the resistance to UV degradation of WBPU coatings. The dispersion of the coating was prepared by a *f*-OFA-CeO_2_ pre-mixing process, using three monomers: PTMG, PDMS and H_12_MDI. Only PDMS pre-mixing favored *f*-OFA-CeO_2_ distribution on the top of the surface to enhance the resistance to UV degradation. The coating was only slightly degraded following sunlight exposure for 8 months. The hydrophobicity of this coating also dramatically changed which is another advantage of this outdoor coating. Such types of functionalized waste material can also be considered for other popular coatings. Though we used this coating on a mild steel panel, the formulation can also be applied to other substrates/structures, such as wood, concrete and other metals. This technology could offer new opportunities and uses for local OFA waste material.

## Figures and Tables

**Table 1:** Sample designation and composition of coatings.

Coating	Composition (Mol)						CeO_2_ (2.0 wt%) Mixed Monomer	Hardness			Water Contact Angle			
	PTMG	PDMS	DMPA	TEA	EDA	H_12_MDI								
WBPU-PTMG	0.730	…	0.920	0.920	0.350	2.000	…	69	±	1	64		±	0.5
WBPU-PTMG-Ce	0.730	…	0.920	0.920	0.350	2.000	PTMG	81	±	1	95	±		0.5
WBPU-PTMG-NCO-Ce	0.730	…	0.920	0.920	0.350	2.000	H_12_MDI	81	±	1	93	±		1.0
WBPU-PDMS	0.300	0.430	0.920	0.920	0.350	2.000	…	71	±	1	84	±		1.0
WBPU-PDMS-Ce	0.300	0.430	0.920	0.920	0.350	2.000	PDMS	82	±	1	103	±		0.5
WBPU-PDMS-NCO-Ce	0.300	0.430	0.920	0.920	0.350	2.000	H_12_MDI	82	±	1	97	±		1.0

## Data Availability

Not applicable.
